# Characterization of Pulse-Containing Cakes Using Sensory Evaluation and Instrumental Analysis

**DOI:** 10.3390/foods13223575

**Published:** 2024-11-08

**Authors:** Ine Heetesonne, Elke Claus, Ingrid De Leyn, Koen Dewettinck, Melissa Camerlinck, Joachim J. Schouteten, Filip Van Bockstaele

**Affiliations:** 1 Research Group Food Structure and Function, Faculty of Bioscience Engineering, Department of Food Technology, Safety and Health, Ghent University, Coupure Links 653, 9000 Ghent, Belgium; 2 Research Centre of AgroFoodNature, HOGENT University of Applied Sciences and Arts, Valentin Vaerwyckweg 1, 9000 Ghent, Belgium; melissa.camerlinck@hogent.be; 3 Research Unit of Cereal and Feed Technology, Faculty of Bioscience Engineering, Department of Food Technology, Safety and Health, Ghent University, Valentin Vaerwyckweg 1, 9000 Ghent, Belgium; 4 Department of Agricultural Economics, Ghent University, Coupure Links 653, 9000 Ghent, Belgium

**Keywords:** pulses, sensory, cake, E-nose, legumes

## Abstract

Despite the nutritional and environmental benefits of pulses, their incorporation into bakery products has been impeded by their characteristic off-flavour. This study characterizes five pulses (faba bean, chickpea, whole lentil, split pea and pinto bean) in a cake application with a 40% wheat flour substitution, alongside a control cake. Physicochemical analysis and sensory analysis using a consumer panel (n = 124) and instrumental analysis (GC E-nose) were conducted. The liking scores for the pulse-containing cakes were significantly lower compared to the control cake, but half of the participants preferred a pulse-containing cake, indicating their market potential. Both instrumental analysis and sensory evaluation identified the chickpea and faba bean cakes as most similar to the control, while the pea cake was the most divergent. This cake was described as beany and grassy by consumers, negatively affecting the overall acceptance. Consumers in the sensory study had difficulties in distinguishing between the chickpea and faba bean cakes. Similarly, based on the volatile profiles, the chickpea and faba bean cakes demonstrated the closest relationship. The alignment between sensory data and E-nose results supports the added value of instrumental techniques such as the GC E-nose in sensory research.

## 1. Introduction

Pulses, the edible seeds of plants within the Leguminosae family, are a staple food for a significant portion of the world’s population. They have an interesting nutritional profile, with a high protein (21–25%) and fibre (12–20%) content. They are a valuable source of minerals such as iron, zinc and magnesium, and contain phytochemicals with antioxidant and anticarcinogenic effects [1,2,3]. In addition to their nutritional benefits, pulses offer environmental and socio-economic advantages. This leads to rising interest in adding pulses to formulated food products, as consumers are increasingly environmentally and health-conscious [4]. 

In bakery products, adding pulses and pulse ingredients could improve protein quantity and quality [5]. They provide lysine, an amino acid often limiting in cereals, while cereals are higher in sulphur-containing amino acids such as methionine and cysteine, and tryptophan [6,7]. The influence of the addition of pulses on the nutritional and functional quality of bakery products has been well studied. In gluten-containing bread, the partial replacement of wheat flour with pulse flour results in bread with higher protein and fibre content, and lower gluten and starch content [5,8]. The decrease in gluten, competition between wheat and pulse proteins, and fibre interactions cause an impaired gluten network formation. This could lead to bread with lower volume, and poorer structure and texture [9,10]. Therefore, only low percentages (10–15%) of flour replacement are generally applied in bread-making [5,11]. Additionally, higher amounts of pulse flour lead to a darker bread, as more lysine increases the Maillard reaction [9].

Unlike bread, cake does not require the development of a gluten network to obtain its structure, allowing for higher substitutions of wheat flour with pulse flour without significantly losing structure [12,13,14]. However, as pulse flour has three times the protein content and half the starch content compared to cake flour, the reduced starch-to-protein ratio could cause insufficient stabilisation of the gas cells during batter solidification, causing denser cakes [15,16]. Fibres may also influence the organisation of the starch–protein network, leading to a consequent reduction in cake volume [13]. This reduction in volume and increase in hardness can lead to reduced consumer acceptance, and has been proven in cakes containing 50% chickpea flour [13], 25% pea flour [12], 50% lentil flour [14] and, respectively, 25% and 20% pea pod flour and faba bean pod flour [17]. 

One of the major obstacles for the incorporation of pulses is their typical off-flavour profile, which may affect the sensory properties of the products they are added to. These off-flavours originate from volatile and non-volatile compounds that are either inherent to the pulse or develop during processing and storage (mainly lipid oxidation and amino-acid degradation compounds) [18]. The term ‘beany’ is commonly used to describe the aroma and flavour (perceived through orthonasal and retronasal olfaction) of pulses. This term encompasses attributes such as green, earthy, nutty and grassy [19,20,21]. 

Khrisanapant et al. [3] compared the volatile compounds found in the headspace of different pulses using GC-MS. They found the most abundant functional group to be the aldehydes, with hexanal being the most detected compound in all pulses except for the navy bean. The alcohols were the second most abundant functional group with 1-hexanol representing between 4.4 and 16.4% of the volatiles in chickpeas, faba beans, lentils and peas. Other prominent alcohols in the pulses were 1-penten-3-ol, 1-octen-3-ol and 3-methylbutanol [3]. Similarly, Badjona et al. [22] assigned the compounds mentioned above as major off-flavours in faba beans. They used the literature to describe hexanal as cut-grass and green, hexanol and 1-penten-3-ol as beany and green, 1-octen-3-ol as mushroom and 3-methylbutanol as balsamic.

Despite the sensory characteristics of the native pulse ingredients being well researched, the effect of their addition to the sensory profiles of (bakery) products is less explored in academic papers. Krause et al. [16] characterized the volatiles in sponge cakes and batters containing 100% pulse flour. Cakes containing yellow and green pea flour had significantly higher concentrations of lipid oxidation (catalyzed by lipoxygenase) markers (alcohols, aldehydes, ketones), which typically enhance green-beany flavours, while the chickpea and lentil cakes showed more Maillard markers (pyrazines and furanic compounds), associated with nutty, roasted flavours. However, no consumer study was performed. Drakula et al. [23] investigated the volatile profile and sensory properties (trained panel, n = 13) of gluten-free bread containing yellow pea flour. They found that, compared to the control gluten-free bread, the version with 25% yellow pea flour had higher concentrations of alcohols (e.g., 1-pentanol, 1-hexanol, 1-octen-3-ol) and aldehydes (e.g., hexanal, pentanal, (E)-2-octenal). The trained panel noted an increased pea flavour, dusty/musty flavour and pea aftertaste, leading to a decreased hedonic score. Comparably, cakes made by Belghith-Fendri et al. [17], containing pea pod flour and faba bean pod flour (0% to 30% pulse pod flour in increments of 5%), were evaluated by an untrained panel (n = 40). The flavour of all cakes containing pea and faba bean pod flour was liked significantly less than the control. The liking score decreased with each increase of pulse pod flour. Similar results, in which the addition of pulse flour to bakery products leads to a decrease in liking or acceptance have been found in cookies [24,25] and breads [26,27,28].

This study aimed to gain more insight in the sensory profile of pulse flour-containing pound cake. Five commercially available pulse flours (faba bean, chickpea, whole lentil, split pea and pinto bean) were incorporated in a model pound cake recipe at a wheat flour substitution rate of 40%. Sensory profiling was performed by instrumental analysis (E-nose) and through a consumer study which, to our knowledge, has not been reported previously. Next to investigating the impact of the pulse flour type on the sensory profile of the cakes, the secondary goal was to investigate the suitability of the fast E-nose methodology for sensory profiling of pulse-containing foods. 

## 2. Materials and Methods

### 2.1. Materials

Epi B wheat flour (WF) was purchased from Paniflower (Merksem, Belgium). Sodium pyrophosphate was purchased from Ingrizo (De Pinte, Belgium). All other ingredients such as fine sugar, eggs, margarine and sodium bicarbonate were obtained from the local market. Dry pulses were purchased online (peulvruchten-bestellen.nl) and processed into flour using a FRITSCH Pulverisette 14 rotor mill (Pittsboro, NC, USA). The following pulses were used: pinto bean (*Phaseolus vulgaris*), chickpea (*Cicer arietenum*), dehulled faba bean (*Vicia faba*), dehulled (split) green pea (*Pisum sativum*) and whole red lentil (*Lens culinaris*). They are referred to as FB = faba bean; CP = chickpea; WL = whole lentil; SP = split pea; PB = pinto bean.

### 2.2. Cake-Making Procedure

The recipe and procedure were based on a standard pound cake recipe [29]. One batch of batter consisted of 300 g wheat flour, 300 g fine sugar, 300 g beaten egg, 300 g margarine, 3.43 g sodium pyrophosphate and 2.57 g sodium bicarbonate. In the cakes containing pulse flour, 40% of the wheat flour was substituted with the same weight of pulse flour. Four cakes per formulation were produced for analysis.

The cake batter was produced using a multi-stage mixing method. Sugar and margarine were creamed for 3 min at speed 2 on a Hobart N-50 electric mixer with a flat beater attachment (St. Joseph, MI, USA). The sieved flours and baking powders were added in alternation with the beaten eggs at speed 1. This was mixed for 3.5 min. Before finishing, the edges were scraped and the batter was mixed for another 30 s. Then, 250 g of batter was transferred to aluminum baking tins (18.9 × 8.8 × 5.2 cm) and baked at 175 °C for 35 min in a MIWE Aero FP12 convection oven (Arnstein, Germany). After 1.5 h of cooling down, the cakes were packed in plastic bags. All physicochemical and sensory tests were performed one day after baking. 

### 2.3. Physicochemical Properties

The cake batter density was measured in quadruplicate at room temperature by measuring the mass of batter in a cylindrical cup of known volume. The density was calculated by dividing the weight of batter by its volume and expressed as kg/m^3^. 

The volume of the cakes after baking was measured in quadruplicate at room temperature using a laser scanning system, the Volscan Profiler 600 (Stable Micro Systems Ltd., Vienna Court, Godalming, UK). The laser step was set to 5 mm with a rotation speed of 1.5 rps. By dividing the mass by the volume, the specific volume of the cakes was calculated.

The water activity of the cake crumb was measured in triplicate using the LabMaster aw-meter (Novasina AG, Lachen, Switzerland). 

Crust and crumb colour were measured using a spectrophotometer CM700d (Konica Minolta, Tokyo, Japan) to determine L* (darkness to lightness; 0 to 100), a* (greenness to redness; −60 to +60) and b* (blueness to yellowness; −60 to +60). The overall colour difference (Δ*E*) between the sample and the control was calculated as $\Delta E=\sqrt{(\Delta L^{*})2+{\left(\Delta a^{*}\right)}^{2}{+\left(\Delta b^{*}\right)}^{2}}$. These measurements were done six times per sample. For the visualization of the crumb structure, scans were taken of the middle slice using a CanoScan LiDE 300 scanner (Canon, Tokyo, Japan). 

The cake crumb texture was measured using a TA.XTplus texture analyser from Stable Microsystems (Vienna Court, Godalming, UK) with a 5 kg load cell and a cylindrical probe (Ø 35 mm, P/35P). The probe made two compressions (40% strain, test speed 1.70 mm/s) on a cake slice of 2.5 cm thickness to imitate a chewing motion. By this analysis, cake hardness was assessed. This measurement was done six times per sample.

### 2.4. Fingerprinting of Volatile Compounds

Fingerprinting of volatile compounds was performed using an ultra-fast GC electronic nose (Heracles II, Alpha M.O.S., Toulouse, France), equipped with an autosampler (Odour Scanner HS 100, Alpha M.O.S., Toulouse, France), a cooled Tenax trap, two capillary columns of different polarities and two flame ionization detectors (FIDs). The two parallel columns were the non-polar MXT-5 (5% diphenyl) and a medium polar MXT-1701 (14% cyanopropylphenyl) column with dimensions of 10 m × 180 μm × 0.4 μm. 

Prior to analysis, 2 g of cake was weighed in 20 mL vials, which were then tightly capped with PTFE/silicone seals to prevent the penetration of environmental odours. Each cake sample was analysed five times and blanks (empty vials) were added before, in between and after the measurements of different samples. To introduce the volatile compounds into the headspace, vials were incubated for 20 min at 60 °C with a constant agitation of 500 rpm. After incubation, the headspace was collected into a syringe of 70 °C at a speed of 500 µL/s. Then, 5 mL was injected into the GC system for 45 s at 125 µL/s at 200 °C and 10 kPa with a hydrogen N7.0 carrier gas (Parker Balston, Gateshead, Tyne & Wear, UK). Afterwards, a trap system with an initial condition of 40 °C for 50 s was heated in 35 s to 240 °C and sustained for 30 s. The vent rate of the injector was at 30 mL/min and the split of the trap at 10 mL/min. At a temperature of 250 °C, the valve sent the volatiles to the columns. The oven had an initial temperature of 50 °C for 2 s, whereafter it was raised to 80 °C at 1.0 °C/s and immediately ramped up to 250 °C at 3.0 °C/s and kept for 21 s. The temperature of the FIDs was 260 °C, and the gain and offset of both FIDs were 12 and 1000, respectively. The total acquisition time was 110 s with an acquisition period of 0.01 s [30].

### 2.5. Sensory Evaluation

#### 2.5.1. Sample Preparation

All cakes were prepared the day before the consumers’ tests took place. Respondents were served five slices of cake containing pulse flour and one control cake, all freshly sliced and served in plastic see-through containers foreseen with a random three-digit code. They received the cakes along with a transparent odourless cup of water. Samples were evaluated according to an experimental design that was balanced for order and carry-over effects [31]. The respondents filled in an online evaluation questionnaire using the EyeQuestion 5.4.7 software (Logic8 BV, Elst, The Netherlands) in the sensory lab facilities of Ghent University at 21 °C with artificial lighting. 

#### 2.5.2. Participants

A total of 123 participants were recruited at a university campus in Ghent, Belgium, through flyers in May 2023. Only product users without allergies (gluten-containing grains, wheat, milk, egg, pulses) or taste/odour deficiencies were allowed to participate. 

The participants comprised young adults and university staff volunteers, of which 77 were women and 46 men. The mean age of the participants was 25 ± 7 years. All individuals provided informed consent, and ethical approval (reference number: 2023–13) was provided by the ethical committee of the Faculty of Political and Social Sciences (Ghent University). 

#### 2.5.3. Experimental Procedure

The online questionnaire started with a description of the test, a declaration of consent, questions for participant compatibility and questions about how often they consume cake and/or pulses (1: “daily” to 7: “never”) [32,33].

The first part of the tasting enquired the assessment of the overall liking score on a 9-point hedonic scale from 1: “dislike extremely” to 9: “like extremely” [34].

Next, cakes were evaluated on appearance, aroma, flavour and taste and texture by using the check-all-that-apply (CATA) approach. The attributes and samples were given in a randomized order to eliminate primacy bias [35]. The attributes used to describe the cakes were based on the literature [20,21,36] and relevance by the involved researchers. These are presented in Table 1.

Next, participants indicated their favourite cake and selected the attributes that defined their ideal cake [37,38].

Participants were also asked 6 questions of the Food Neophobia Scale (FNS) [39] and 10 questions of the Food Choice Questionnaire (FCQ) [40], both on a 7-point Likert scale (1: strongly disagree to 7: strongly agree) (Table 2). Finally, they provided their gender and age.

### 2.6. Statistical Analysis

Statistical analyses on physicochemical measurements were performed with SPSS Statistics 27 (SPSS Inc., Chicago, IL, USA). All tests were done at a significance level of 0.05. One-way ANOVA was used to investigate any significant difference between the samples. Testing for equal variances was executed with the Levene Test. When conditions for equal variance were fulfilled, the Tukey test was used to determine differences between samples. In case variances were not equal, the Games–Howell test was performed. If data were not normal, a Kruskal–Wallis test was used for multiple pairwise comparison. All results are displayed as mean ± standard deviation. 

The data obtained by the GC E-nose were analysed within the AlphaSoft software (v12.4, Alpha M.O.S., Toulouse, France). The Discrimination Index (DI) is a parameter created by the software, and can be used to describe the discriminatory power of the groups’ separation. Alpha MOS considers that good discrimination and reliability are achieved for DI over 80%. This parameter is purely explorative, without providing statistical significance. Further, the chromatograms recorded by the GC E-nose were analysed by principal component analysis (PCA) and distance analysis to visualize natural clustering in the data. Automatic data reduction was used and only sensors with a discrimination power higher than 0.9 were selected. Statistical differences in the peak areas of the chromatographic peaks were determined using one-way ANOVA in SPSS Statistics 27 (SPSS Inc., Chicago, IL, USA). 

Statistical analysis for the consumer study was executed in XLSTAT 2023 (Addinsoft, New York, NY, USA) with the sensory package. Cochran’s Q tests were used to test the effect of explanatory attributes. For significant values, a multiple pairwise comparison according to the Sheskin procedure was executed. Differences in overall liking were determined by one-way ANOVA. A penalty analysis was performed to identify which CATA attributes had the highest impact on hedonic consumer liking [37,41].

## 3. Results

### 3.1. Physicochemical Properties

The physicochemical properties of pound cakes containing 100% wheat flour (control) and 40% pulse flours are presented in Table 3. The specific volume of the FB cake (2.03 ± 0.02 mL/g) was significantly higher compared to the control cake (1.94 ± 0.04 g/mL). In terms of texture, only the PB cake was significantly harder than the control cake. No significant differences were found between the cakes for water activity or batter density. 

**Figure 1:**
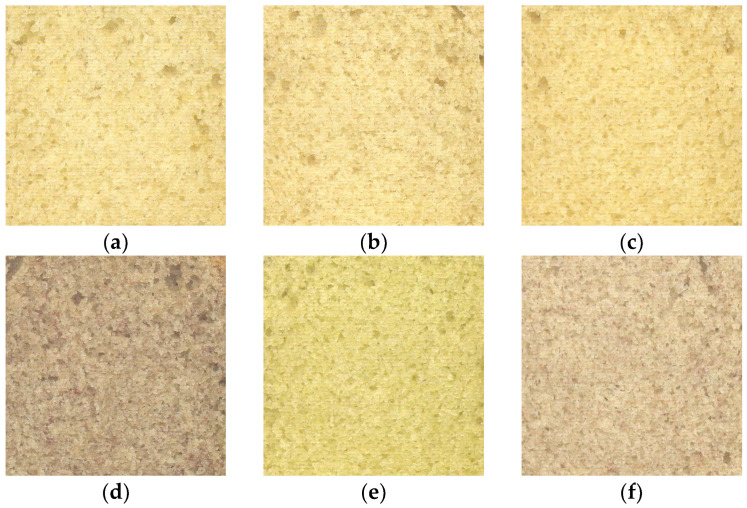
Scans (50 × 50 mm) of crumb structure of pulse-containing cakes ((**a**) = control; (**b**) = faba bean; (**c**) = chickpea; (**d**) = whole lentil; (**e**) = split pea; (**f**) = pinto bean).

Partial substitution of wheat flour by pulse flour changed the colour of the crumb and, to a lesser extent, that of the crust. The CP and FB cakes resembled the control the most on all dimensions. Visually, these were very similar (Figure 1). The cakes containing pinto bean (PB) and lentil flour (WL) were the darkest, and the pea flour cake (SP) was the greenest, with a negative a*-value of −1.65.

### 3.2. E-Nose Fingerprinting

Using the Heracles GC E-nose, chromatograms of the volatile compounds in the headspace of the cakes could be obtained. These chromatograms are provided in the Supplementary Materials (Figures S1–S12). The areas of the relevant peaks were used to generate specific odour fingerprints for each sample. 

**Figure 2:**
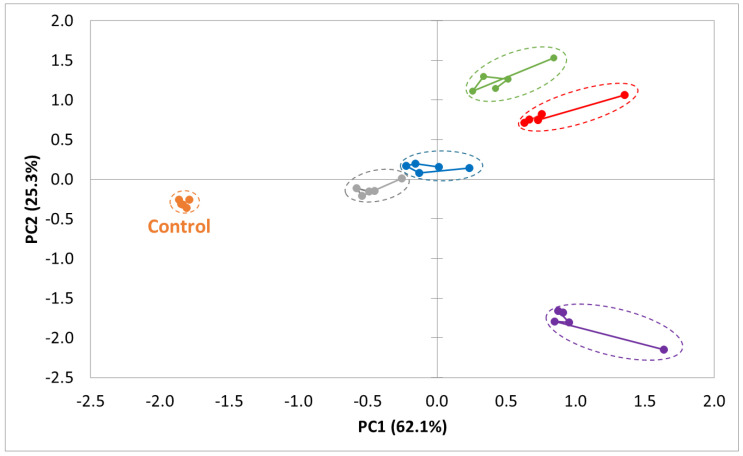
PCA plot of the volatile fingerprints of pulse-containing cakes (analysed by ultra-fast GC E-nose) (control = orange; FB (faba bean) = grey; CP (chickpea) = blue; PB (pinto bean) = green; WL (whole lentil) = red; SP (split pea) = purple).

Of the 100 captured sensors, 21 exhibited a discrimination power greater than 0.9. Principal component analysis (PCA) was conducted using these 21 sensors to reduce the number of components while preserving essential information. This data analysis enabled the visualisation of natural clustering among the cake samples (Figure 2). The first two principal components accounted for 87.40% of the total variability. The discrimination index was 86, indicating that all pulse cakes could be distinctly differentiated.

The PCA plot shows the control cake in the third quadrant. The FB and CP cakes are close together, near the centre of the plot, while the PB and WL cakes are located in the first quadrant. The SP cake is located in the fourth quadrant. 

The compounds predominantly responsible for discriminating the samples (sensors with PC loadings > 0.90) and their peak areas for each cake are included in the Supplementary Materials (Table S1). However, the experimental Kovats Indices (KI_exp_) were not linked to the literature Kovats Indices (KI_lit_), as identification using the literature and the AroChemBase yielded inconclusive results. Furthermore, identification using an ultra-fast GC E-nose is only tentative, and other techniques such as GC-MS should be used for this purpose [30]. 

A distance analysis was performed to show similarities and differences between samples based on their volatile profiles (Table 4) resulting in a distance and pattern discrimination index between samples. All groups showed a high discrimination index, indicating good differentiation between the cakes. 

The cakes with the most similar volatile profile (closest distance) were the CP and FB cakes (2.80) and the PB and WL cakes (4.34). The cake most different from the control cake in terms of volatile profile was the SP cake (11.16). 

### 3.3. Consumer Study

#### 3.3.1. Liking and Preference

Table 5 presents the mean liking scores and preferences for the control cake and the cakes made with 40% pulse flour. Overall, the control cake was ‘liked moderately’. FB, CP, WL and PB were rated between ‘neither liked nor disliked’ and ‘liked slightly’. SP had the lowest liking score, between ‘neither liked nor disliked’ and ‘disliked slightly’. 

Of the 123 participants, 46.3% preferred the control cake without pulse flour. SP was preferred the least (2.4%), followed by PB (10.6%). FB, CP and WL were each preferred by 13.0 to 13.8% of participants.

#### 3.3.2. Sensory Characterization by CATA Analysis

##### Appearance

Table 6 displays the frequency of mention (%) of each attribute related to the appearance of the cakes using the CATA method. The attributes dark crust, reddish and speckled were cited more frequently for the cakes containing WL and PB flour. The SP cake was distinguished by its greenish colour, while the control, FB and CP cakes were viewed as yellowish. SP, WL and PB were also described as having an unnatural appearance, which had a negative correlation (*p* = −0.759) with yellowish.

##### Taste, Flavour and Aroma

Out of twenty attributes related to flavour, taste and aroma, four did not differ among the cakes, according to a Cochran’s Q test: sour aroma (*p* = 0.540), bland aroma (*p* = 0.216), bitter flavour (*p* = 0.115), sour flavour (*p* = 0.476). These attributes were excluded from further analysis. Table 7 shows the frequency of mention for all other attributes of flavour, taste and aroma.

The cake containing split pea flour (SP) was characterized by a significantly higher beany and grassy aroma and flavour compared to all other cakes. The PB and WL cakes were perceived as nutty by around half of the participants. 

The vast majority described the control cake as buttery, with only the FB cake being significantly indifferent for buttery aroma. FB and CP received higher scores for buttery aroma and flavour compared to the other pulse-containing cakes. 

More than half (56.1%) of the participants perceived the control cake as having a vanilla aroma, which was significantly higher than the pulse cakes. Similarly, cakes containing pulse flour were perceived as significantly less sweet compared to the control. 

More than 35% of participants described the WL, SP and PB cakes as earthy, contrasting sharply with 2.4% for the control cake. Additionally, the control had a good aftertaste and sweet taste.

##### Texture

A Cochran’s Q test showed that the attributes sticky (*p* = 0.617), chewy (*p* = 0.125) and crispy crust (*p* = 0.122) did not differ among the cakes. These attributes were excluded from further analysis. Table 8 presents the frequency of mention for all other texture attributes.

The control cake was perceived as the least dry and most moist, with only the FB being significantly indifferent. The WL and PB were noted to be more granular than the control. 

#### 3.3.3. Drivers of Liking

The participants were asked to select the attributes that applied to the control cakes and the five cakes containing pulse flours. In addition, they had to check the attributes that they believed applied to an ideal cake. These data were used for a penalty analysis to determine drivers of liking [37], presented in Table 9. Of the 28 attributes used to describe and characterize the cakes, 9 were identified as ‘must have’. These attributes significantly enhance overall liking when present in both the ideal and real product [38,42]. 

The attributes of bland flavour, long aftertaste and nutty aroma and flavour do not harm the overall liking of the cakes. However, a bad aftertaste, an earthy flavour and a beany aroma and flavour do harm the acceptance. Together with a dry and granular texture, they are ‘must not have’ attributes.

**Figure 3:**
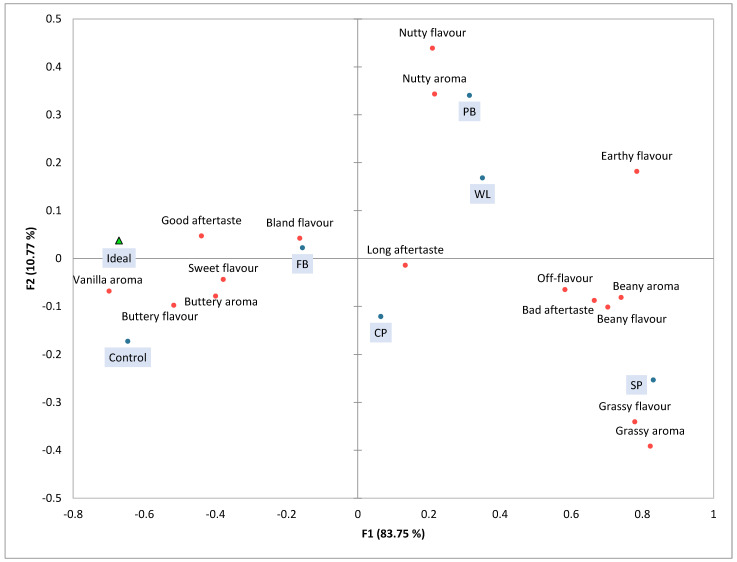
Correspondence analysis (CA) plot of taste, flavour and aroma attributes (orange dots) for pulse-containing cakes (blue dots) and an ideal cake (green triangle).

In Figure 3, the correspondence analysis (CA) plot of the attributes of taste, flavour and aroma are presented. The two dimensions explained 94.52% of the total variance, with the first dimension explaining 83.75% and dimension 2 explaining 10.77%. Given the high variance explained by D1, the plot could be interpreted as almost unidimensional. This highlights the close resemblance between the control cake and the ideal cake, which are associated with attributes such as good aftertaste, sweet flavour, buttery flavour and aroma and vanilla aroma. The PB and WL cakes were grouped close together. They are associated with a nutty flavour and aroma. The SP cake is furthest removed from the control and ideal cakes, and is related to attributes such as grassy, beany, off-flavour and bad aftertaste. 

## 4. Discussion

In the present study, the substitution of 40% wheat flour with different pulse flours was investigated in a pound cake application, considering physicochemical and sensorial properties. 

When comparing a control pound cake (100% wheat flour) to pound cakes made with a 40% substitution of pulse flours (faba bean, chickpea, whole lentil, split pea and pinto bean), the physicochemical properties differ minimally. The cake made with faba bean flour had a significantly higher specific volume compared to the control cake, but did not differ from the other pulse cakes (*p* < 0.05). Prior studies showed the opposite result, where the substitution of wheat flour for pulse flours in a cake application reduced the volume [13,14,17]. The cake made with pinto bean flour (PB) was significantly harder compared to the control. However, an increase in hardness from 987 g (control) to 1169 g (pinto bean) would be unnoticeable to the average consumer. The observed differences (although limited) in physicochemical attributes may have been caused by deviations in the moisture content or granulation of the pulse flours. 

A great distinction between the cakes was their crumb colour, measured using the CIELab colour scale. The cake made with split pea flour (SP) had a distinct green hue, while the whole lentil (WL) cake and PB cake were the darkest, exhibiting a red hue. Their crumb colour differed the most from the control, while the faba bean (FB) cake and the chickpea (CP) cake were most similar in crumb colour to the control. The sensory panel noted similar differences, describing the SP cake as greenish and unnatural, the WL and PB cakes as reddish and speckled, and the control, FB and CP cakes as yellowish and least unnatural. Despite the speckled and reddish attributes not harming the overall liking of the cakes in our study, a yellowish colour was deemed essential (must have attribute). This confirms the importance of colour as a critical parameter for the acceptance of cake, as supported by the literature [43].

The volatiles present in the headspace of the cakes were analysed using the Heracles GC E-nose. This device generated chromatograms based on the volatile compounds present in the headspace of the samples. By performing a principal component analysis and a distance analysis, the relationships between the volatile profiles of the cakes could be established. The discrimination powers show a high statistical difference between the cakes. The SP cake had the most divergent volatile profile compared to the control cake. The two cakes with the most similar volatile profiles were the CP and FB cakes. They were also the closest related to the control cake. Krause et al. [16] used instrumental analysis (GC-MS) to characterize the volatiles in cakes containing 100% wheat flour and 100% pulse flour. They found the pea cakes to be most different from all other cakes (chickpea, lentil). This was accounted to the higher amounts of lipid oxidation markers (alcohols, aldehydes, ketones). The chickpea and lentil cakes showed more Maillard markers (pyrazines and furanic compounds). No cakes with bean or faba bean flour were included in their study. 

In addition to analysing the control and pulse-containing cakes using an E-nose, they were evaluated and characterized through a consumer study. This showed that, despite receiving a significantly higher liking score, the control cake was preferred by only 46.3% of consumers. This means that more than half of the participants preferred a cake containing pulse flour, indicating the potential for acceptance when targeting specific consumer segments. The SP cake had the lowest liking score and was preferred by only 2.4% of participants. Participants characterized this cake as significantly more grassy and beany compared to all other cakes. A similar result was found in a study by Frohlich et al. [44], where pita breads made with 30% pea flour exhibited a stronger beany flavour compared to breads made with navy bean or faba bean flour. A penalty analysis classified a beany aroma and beany flavour as ‘must not have’ attributes, meaning their presence reduces the acceptance of the cakes in our study. 

Around half of the participants perceived the PB and WL cakes as nutty. A nutty flavour was also perceived in brownies made with 100% bean flour [45], and in a cake made with 26% of a lentil protein solution (10 g/100mL) [46]. In the last study, the nutty flavour was perceived as favourable by the sensory panel. In our study, a nutty flavour was seen as not harmful to the liking of the cakes. 

The vast majority of participants described the control cake as buttery, with only the FB cake being significantly indifferent for a buttery aroma. FB and CP cakes received higher scores for buttery aroma and flavour compared to the other pulse-containing cakes. 

Even though no vanilla was added to any of the cakes, more than half (56.1%) of the participants perceived the control cake as vanilla-like, which was significantly higher than the pulse cakes. This could be due to the assumption made by consumers that a standard pound cake must contain vanilla flavouring. However, no strong correlations were found between vanilla aroma and yellowish (*p* = 0.443), sweet flavour (*p* = 0.554), buttery aroma (*p* = 0.280) or buttery flavour (*p* = 0.373). Similarly, cakes containing pulse flour were perceived as significantly less sweet compared to the control, despite using the same amount of sugar. This reduction in perceived sweetness aligns with previous research in chocolate brownies [45] and in cookies [47], both containing bean flour. Sparvoli et al. [47] attributed these findings to a reduced sucrose sensitivity due to a higher umami flavour in bean-containing products. 

The exact explanation behind the perceived intensity of attributes such as a buttery flavour and aroma, vanilla aroma and sweet taste deserves further research, as these attributes positively influence the overall liking of the cakes in our study. Other ‘must have’ attributes are a spongy and moist texture, which was confirmed in the penalty analysis performed by Jarpa-Para et al. [46] for cakes containing lentil protein. 

Based on the consumer study, it can be concluded that the control cake closely resembles consumers’ idea of an ideal cake, which needs to be yellow, spongy, moist, buttery, sweet and vanilla-like. The CP and FB cakes most closely resemble the control cake, particularly in their yellowish colour, buttery flavour and aroma and lack of earthy flavour. In contrast, the SP cake was the least similar to the control, with a greenish colour and grassy and beany flavour and aroma. This cake was liked and preferred the least among consumers. 

The CA plot from sensory data and the PCA plot from E-nose data show remarkable similarities, indicating a strong alignment between consumer perceptions and instrumental measurements. In both analyses, the chickpea and faba bean cakes were most closely related to the control cake, while the split pea cake was the most divergent. Consumers in the sensory study had difficulty distinguishing between the chickpea and faba bean cakes, as reflected in their similar scores for the aroma, flavour and taste attributes. Similarly, based on the volatile profiles, the chickpea and faba bean cakes demonstrated the closest relationship. The clear differentiation of the split pea cake in both plots underscores the impact of its distinct volatile profile on sensory perception and consumer acceptance. 

In conclusion, this study demonstrates the potential of pulse flours as a partial wheat flour substitute in pound cakes. Chickpea and faba bean flour showed the most potential, as they resembled the control cake most in sensory attributes, volatile profiles and consumer preference. The combined sensory and instrumental analyses provided a holistic understanding of how pulse flours influence cake quality, suggesting pulse-based cakes can be acceptable with further optimization. Future research should explore different substitution levels, other pulse types and the use of pre-processing to improve flavour and colour. These findings are scientifically significant and offer practical applications for the food industry.

This study is limited by the inability of the GC E-nose to distinctively identify volatile compounds. It is therefore not possible to check the correlation between the sensory attributes (i.e., beany, grassy) and responsible compounds. To achieve this, other instrumental techniques such as GC-MS should be utilized. 

## Figures and Tables

**Table 1:** Sensory terms used for check-all-that-apply questions in consumer tests.

Appearance	Aroma	Flavour and Taste	Texture
Dark crust	Buttery aroma	Buttery flavour	Dry
Speckled	Sour aroma	Sweet taste	Sticky
Yellowish crumb	Beany aroma	Bitter taste	Moist
Light crust	Bland	Bland	Spongy
Greenish crumb	Grassy aroma	Off-flavour	Chewy
Reddish crumb	Nutty aroma	Good aftertaste	Granular
Unnatural	Vanilla aroma	Bad aftertaste	Crumbly
	Earthy aroma	Long aftertaste	Crispy crust
		Beany flavour	
		Nutty flavour	
		Earthy flavour	
		Sour taste	
		Grassy flavour	

**Table 2:** Selected questions from the food choice questionnaire [40] and the food neophobia scale [39]. (R) indicates reverse coded.

Food Choice Questionnaire
**It is important to me that the food I eat on a typical day …**
… is healthy.
… is a way of monitoring my mood (e.g., a good feeling or coping with stress’).
… is convenient (in preparing and buying).
… provides me with pleasurable sensations (e.g., texture, appearance, smell and taste).
… is natural.
… is affordable.
… helps me control my weight.
… is environmentally friendly.
… is animal friendly.
… is fairly traded.
**Food Neophobic Scale**
I am constantly sampling new and different foods. (R)
I do not trust new foods.
If I do not know what is in a food, I will not try it.
At dinner parties, I will try a new food. (R)
I am afraid to eat things I have never had before.
I will eat almost anything. (R)

**Table 3:** Physicochemical properties of cakes with 40% replacement of wheat flour by pulse flours (FB = faba bean; CP = chickpea; WL = whole lentil; SP = split pea; PB = pinto bean).

		Control	FB	CP	WL	SP	PB
**Batter density (kg/m^3^)**		1001 ± 40 ^a^	986 ± 30 ^a^	1005 ± 41 ^a^	996 ± 40 ^a^	993 ± 49 ^a^	1024 ± 48 ^a^
**Specific volume (mL/g)**		1.94 ± 0.04 ^b^	2.03 ± 0.02 ^a^	1.98 ± 0.05 ^a,b^	2.00 ± 0.03 ^a,b^	1.97 ± 0.03 ^a,b^	1.96 ±0.03 ^a,b^
**Water activity (-)**		0.900 ± 0.006 ^a^	0.894 ± 0.005 ^a^	0.889 ± 0.001 ^a^	0.896 ± 0.008 ^a^	0.898 ± 0.011 ^a^	0.894 ± 0.005 ^a^
**Crumb colour (-)**	L*	73.14 ± 1.94 ^a^	71.41 ± 0.90 ^a,b^	72.55 ± 0.68 ^a^	56.16 ± 2.11 ^d^	69.41 ± 0.41 ^b^	64.43 ± 0.54 ^c^
	a*	3.412 ± 0.16 ^b^	3.27 ± 0.19 ^b^	3.57 ± 0.08 ^b^	5.63 ± 0.56 ^a^	−1.65 ± 0.15 ^c^	5.52 ± 0.19 ^a^
	b*	29.70 ± 0.67 ^c^	29.80 ± 0.71 ^c^	32.47 ± 0.44 ^b^	21.17 ± 0.63 ^d^	34.75 ± 0.58 ^a^	20.99 ± 0.521 ^d^
	ΔE*	0	1.75	2.84	19.14	8.07	12.50
**Crust colour (-)**	L*	52.87 ± 4.76 ^a^	43.74 ± 4.23 ^a^	44.94 ± 5.98 ^a^	46.91 ± 5.85 ^a^	47.60 ± 6.13 ^a^	45.01 ± 5.60 ^a^
	a*	17.91 ± 0.84 ^a,b^	18.54 ± 0.56 ^a^	18.40 ± 0.38 ^a^	17.11 ± 0.54 ^b,c^	16.31 ± 1.17 ^c^	17.85 ± 0.66 ^a,b^
	b*	34.55 ± 1.82 ^a^	28.44 ± 3.18 ^a^	29.55 ± 4.62 ^a^	29.07 ± 4.42 ^a^	30.63 ± 4.25 ^a^	28.71 ± 3.82 ^a^
	ΔE*	0	11.00	9.40	8.14	6.76	9.80
**Hardness (g)**		987 ± 35 ^b^	1007 ± 53 ^b^	1072 ± 73 ^a,b^	1078 ± 107 ^a,b^	1117 ± 81 ^a,b^	1169 ± 95 ^a^

Different letters in the same row indicate statistically significant differences (*p* < 0.05).

**Table 4:** Distance analysis between the volatile profiles of different pulse-containing cakes (FB = faba bean; CP = chickpea; WL = whole lentil; SP = split pea; PB = pinto bean).

Class 1	Class 2	Distance	Pattern Discrimination Index (%)
Control	WL	10.26	96.92
Control	SP	11.16	96.60
Control	PB	9.49	97.37
Control	FB	5.24	94.15
Control	CP	7.01	97.45
CP	WL	4.69	84.26
CP	SP	6.50	89.17
CP	PB	4.48	86.32
CP	FB	2.80	76.14
FB	WL	5.92	88.58
FB	SP	6.89	89.51
FB	PB	5.04	87.60
PB	WL	4.34	78.22
PB	SP	7.69	90.40

**Table 5:** Mean liking score on a 1–9 Likert scale (1: dislike extremely, 9: like extremely) and preference of pulse-containing cakes (FB = faba bean; CP = chickpea; WL = whole lentil; SP = split pea; PB = pinto bean) (n = 123).

Cake	Liking Score	Preference (%)
Control	6.86 ± 1.37 ^a^	46.3
FB	5.66 ± 1.72 ^b^	13.0
CP	5.70 ± 1.73 ^b^	13.8
WL	5.62 ± 1.74 ^b^	13.8
SP	4.31 ± 1.84 ^c^	2.4
PB	5.44 ± 1.79 ^b^	10.6

Different letters in the same column indicate statistically significant differences (*p* < 0.05).

**Table 6:** Multiple pairwise comparison using the critical difference (Sheskin) procedure (%) of attributes of appearance from check-all-that-apply data from a consumer study on pulse-containing cakes (FB = faba bean; CP = chickpea; WL = whole lentil; SP = split pea; PB = pinto bean) (n = 123).

Attributes of Appearance	Control	FB	CP	WL	SP	PB
Dark crust	12.2 ^b^	26.0 ^b^	21.1 ^b^	73.2 ^a^	27.6 ^b^	67.5 ^a^
Greenish	0 ^b^	2.4 ^b^	0 ^b^	8.9 ^b^	86.2 ^a^	6.5 ^b^
Light crust	69.1 ^a^	52.0 ^b^	57.7 ^a,b^	8.1 ^d^	27.6 ^c^	17.1 ^c,d^
Reddish	3.3 ^b^	4.9 ^b^	2.4 ^b^	22.0 ^a^	2.4 ^b^	18.7 ^a^
Speckled	3.3 ^b^	3.3 ^b^	0.8 ^b^	69.1 ^a^	1.6 ^b^	68.3 ^a^
Unnatural	0.8 ^c^	2.4 ^c^	0.8 ^c^	33.3 ^a,b^	43.9 ^a^	21.1 ^b^
Yellowish	88.6 ^a^	79.7 ^a^	84.6 ^a^	1.6 ^b^	9.8 ^b^	4.9 ^b^

Different letters in the same row indicate significant differences (*p* < 0.05). Underlined values indicate the highest frequency of mention for each attribute.

**Table 7:** Multiple pairwise comparison using the critical difference (Sheskin) procedure (%) of attributes of aroma, flavour and taste from check-all-that-apply data from a consumer study on pulse-containing cakes (FB = faba bean; CP = chickpea; WL = whole lentil; SP = split pea; PB = pinto bean) (n = 123).

Attributes of Aroma, Flavour and Taste	Control	FB	CP	WL	SP	PB
Beany aroma	3.3 ^c^	15.4 ^b,c^	22.8 ^b^	29.3 ^b^	54.5 ^a^	27.6 ^b^
Buttery aroma	74.8 ^a^	58.5 ^a,b^	50.4 ^b^	28.5 ^c^	22.8 ^c^	30.1 ^c^
Grassy aroma	4.9 ^c^	7.3 ^c^	12.2 ^b,c^	21.1 ^b^	43.9 ^a^	7.3 ^c^
Nutty aroma	12.2 ^c^	28.5 ^b,c^	24.4 ^b,c^	48.8 ^a^	31.7 ^b^	52.8 ^a^
Vanilla aroma	56.1 ^a^	35.8 ^b^	20.3 ^c^	13.0 ^c,d^	4.1 ^d^	13.0 ^c,d^
Bad aftertaste	3.3 ^c^	13.0 ^b,c^	26.0 ^a,b^	17.1 ^b,c^	37.4 ^a^	23.6 ^a,b^
Beany flavour	3.3 ^c^	18.7 ^b^	21.1 ^b^	26.0 ^b^	48.0 ^a^	22.8 ^b^
Bland flavour	13.0 ^a,b^	13.8 ^a^	8.9 ^a,b^	8.1 ^a,b^	3.3 ^b^	8.1 ^a,b^
Buttery flavour	68.3 ^a^	39.8 ^b^	39.8 ^b^	22.0 ^c^	11.4 ^c^	20.3 ^c^
Earthy flavour	2.4 ^b^	12.2 ^b^	11.4 ^b^	35.8 ^a^	45.5 ^a^	35.0 ^a^
Good aftertaste	69.9 ^a^	39.0 ^b^	34.1 ^b^	34.1 ^b^	14.6 ^c^	37.4 ^b^
Grassy flavour	5.7 ^b^	7.3 ^b^	16.3 ^b^	18.7 ^b^	44.7 ^a^	12.2 ^b^
Long aftertaste	14.6 ^b^	16.3 ^a,b^	18.7 ^a,b^	26.8 ^a,b^	30.1 ^a^	17.9 ^a,b^
Nutty flavour	6.5 ^d^	26.0 ^b,c^	17.9 ^c,d^	47.0 ^a,b^	21.1 ^c,d^	45.5 ^a^
Off-flavour	2.4 ^b^	13.8 ^a,b^	14.6 ^a^	13.8 ^a,b^	22.0 ^a^	12.2 ^a,b^
Sweet taste	76.4 ^a^	54.5 ^b^	43.1 ^b,c^	41.5 ^b,c^	23.6 ^d^	29.3 ^c,d^

Different letters in the same row indicate significant differences (*p* < 0.05). Underlined values indicate the highest frequency of mention for each attribute.

**Table 8:** Multiple pairwise comparison using the critical difference (Sheskin) procedure (%) of attributes of texture from check-all-that-apply data from a consumer study on pulse-containing cakes (FB = faba bean; CP = chickpea; WL = whole lentil; SP = split pea; PB = pinto bean) (n = 123).

Attributes of Appearance	Control	FB	CP	WL	SP	PB
Dry	17.9 ^c^	32.5 ^b,c^	38.2 ^a,b^	45.5 ^a,b^	35.8 ^a,b^	51.2 ^a^
Spongy	67.5 ^a^	49.6 ^b^	55.3 ^a,b^	39.8 ^b^	55.3 ^a,b^	40.7 ^b^
Crumbly	19.5 ^b^	23.6 ^a,b^	25.2 ^a,b^	36.6 ^a^	25.2 ^a,b^	30.1 ^a,b^
Moist	53.7 ^a^	39.8 ^a,b^	34.1 ^b,c^	24.4 ^c,d^	26.0 ^b,c,d^	19.5 ^d^
Granular	13.0 ^c^	19.5 ^c^	21.1 ^b,c^	36.6 ^a^	24.4 ^a,b,c^	35.0 ^a,b^

Different letters in the same row indicate significant differences (*p* < 0.05). Underlined values indicate the highest frequency of mention for each attribute.

**Table 9:** Attributes of which the presence influences the overall liking of pulse-containing cakes (drivers of liking).

	Appearance	Aroma, Flavour and Taste	Texture
**Must have**	Light crust	Buttery aroma	Spongy
	Yellowish	Vanilla aroma	Moist
		Buttery flavour	
		Sweet flavour	
		Good aftertaste	
**Does not harm**	Dark crust	Nutty aroma	Crumbly
	Speckled	Bland flavour	
	Reddish	Long aftertaste	
		Nutty flavour	
**Must not have**		Beany aroma	Dry
		Bad aftertaste	Granular
		Beany flavour	
		Earthy flavour	

## Data Availability

The original contributions presented in this study are included in the article and Supplementary Material. Further inquiries can be directed to the corresponding author.

## Supplementary Materials

The following supporting information can be downloaded at: https://www.mdpi.com/article/10.3390/foods13223575/s1, Figures S1–S12: gas chromatogram of cakes obtained by the Heracles II ultra-fast GC E-nose.; Table S1: Peak areas of the sensors most responsible for discrimination between cakes.
