# Supplementary material for: Characterization of Pulse-Containing Cakes Using Sensory Evaluation and Instrumental Analysis
Source: Foods. 2024 Nov 8;13(22):3575. doi: 10.3390/foods13223575 (PMC11592933; doi:10.3390/foods13223575)
Supplement: Supplementary file 1 [file foods-13-03575-s001.zip › foods-3237055-supplementary.pdf]

foods

Supplementary Materials

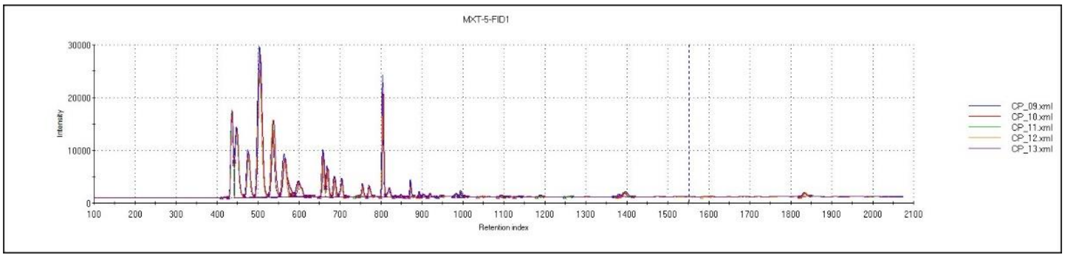

Figure S1: MXT-5 gas chromatogram of chickpea cake (CP) obtained by the Heracles II ultra-fast GC E-nose.

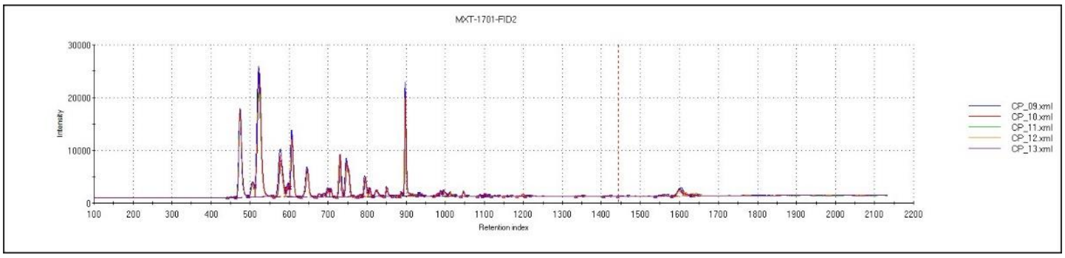

Figure S2: MXT-1701 gas chromatogram of chickpea cake (CP) obtained by the Heracles II ultra-fast GC E-nose

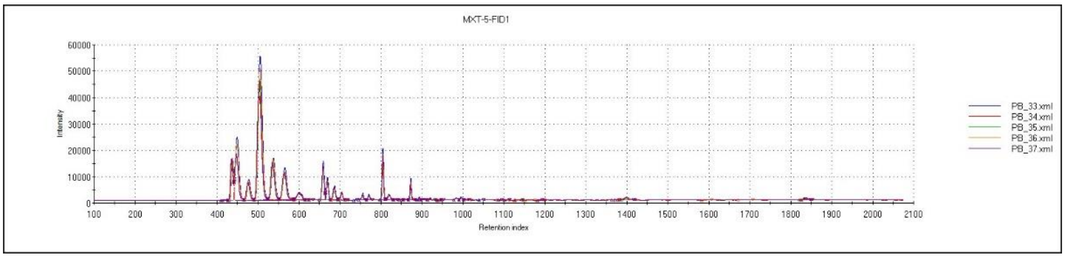

Figure S3: MXT-5 gas chromatogram of pinto bean cake (PB) obtained by the Heracles II ultra-fast GC E-nose.

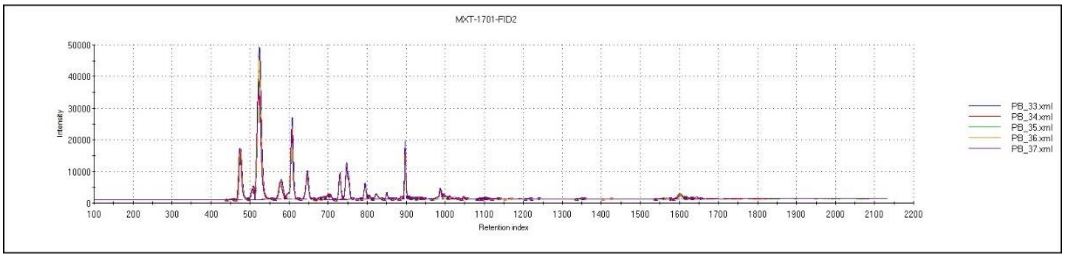

Figure S4: MXT-1701 gas chromatogram of pinto bean cake (PB) obtained by the Heracles II ultra-fast GC E-nose

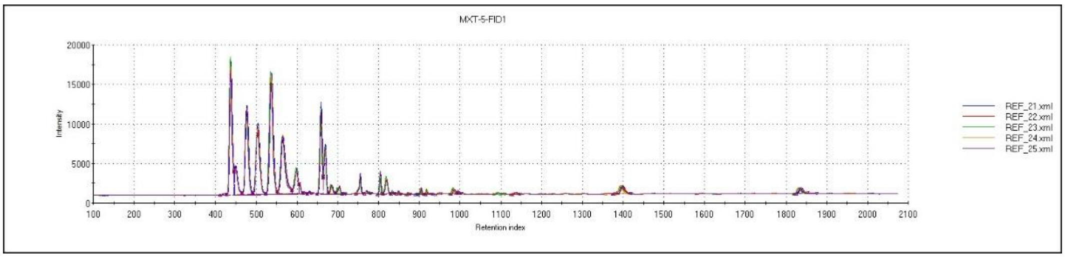

Figure S5: MXT-5 gas chromatogram of control cake (REF) obtained by the Heracles II ultra-fast GC E-nose.

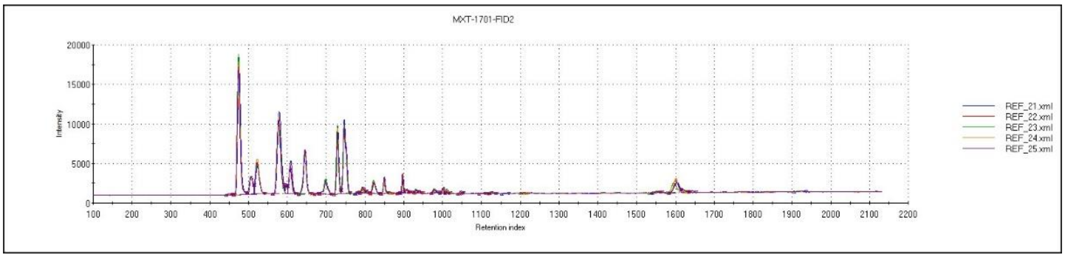

Figure S6: MXT-1701 gas chromatogram of control cake (REF) obtained by the Heracles II ultra-fast GC E-nose.

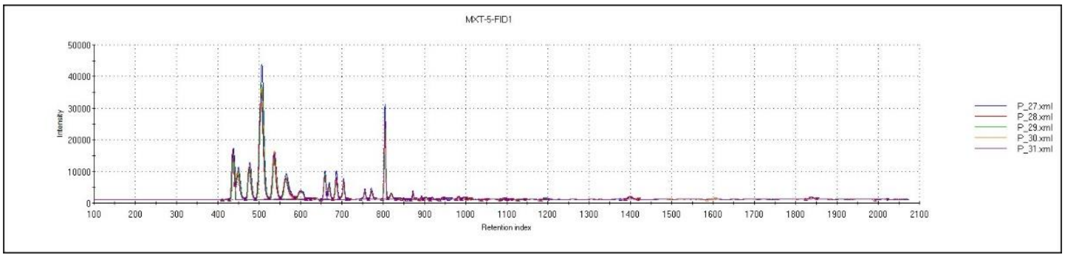

Figure S7: MXT-5 gas chromatogram of split pea cake (P) obtained by the Heracles II ultra-fast GC E-nose.

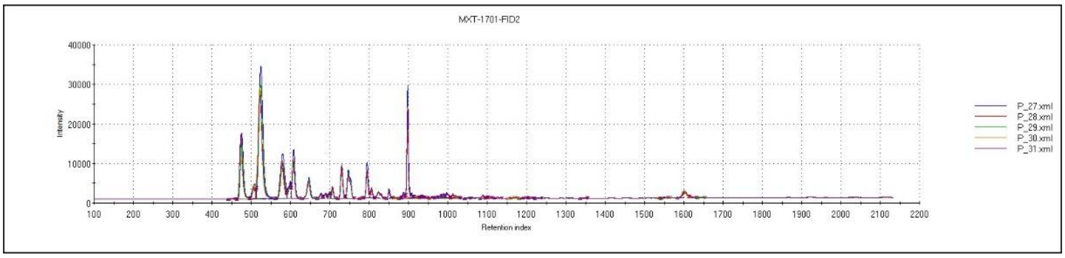

Figure S8: MXT-1701 gas chromatogram of split pea cake (P) obtained by the Heracles II ultra-fast GC E-nose.

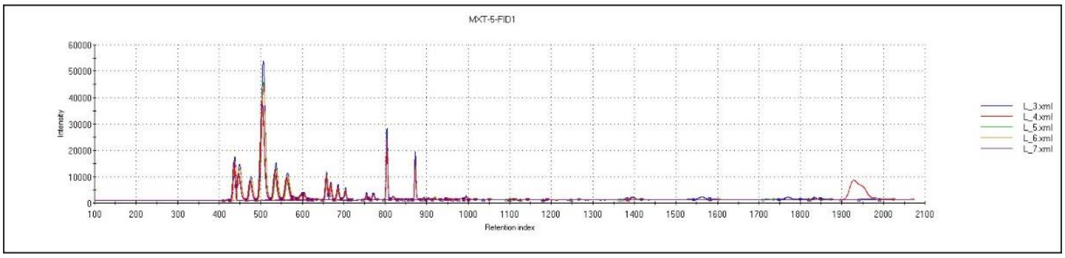

Figure S9: MXT-5 gas chromatogram of lentil cake (L) obtained by the Heracles II ultra-fast GC E-nose.

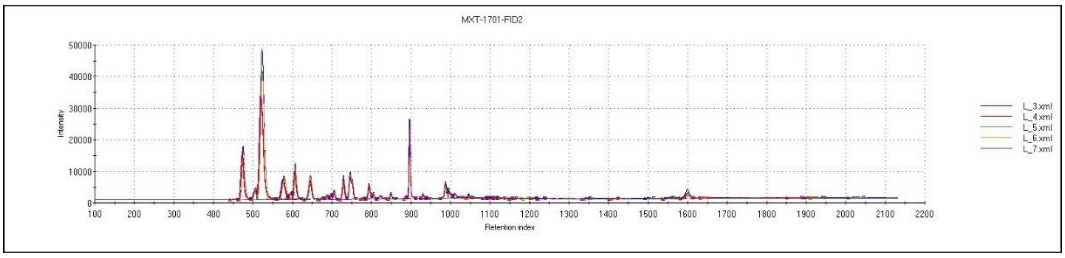

Figure S10: MXT-1701 gas chromatogram of lentil cake (L) obtained by the Heracles II ultra-fast GC E-nose.

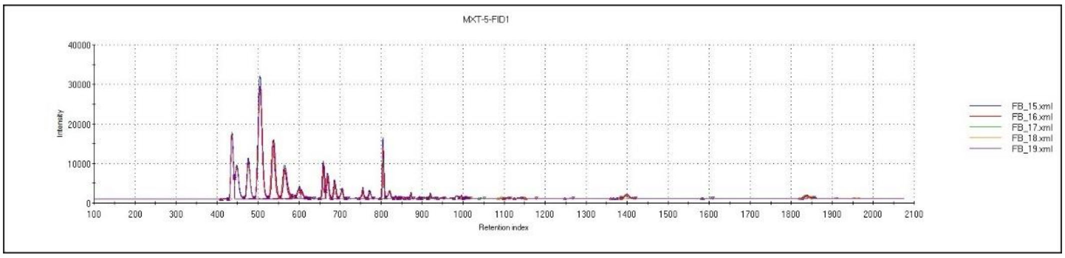

Figure S11: MXT-5 gas chromatogram of faba bean cake (FB) obtained by the Heracles II ultra-fast GC E-nose.

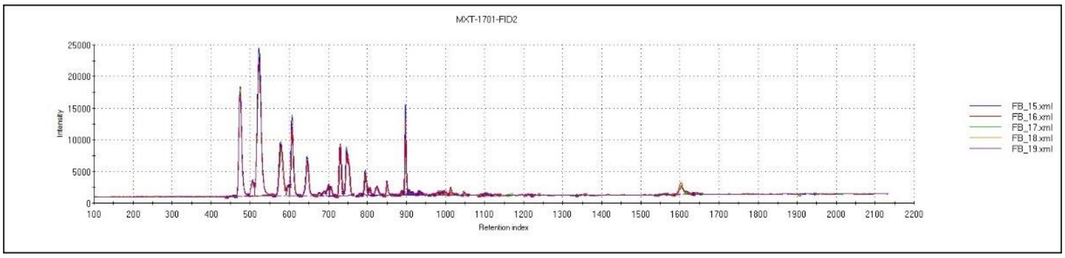

Figure S12: MXT-1701 gas chromatogram of faba bean cake (FB) obtained by the Heracles II ultra-fast GC E-nose.

Table S1: Peak areas of the sensors most responsible for discrimination between cakes (PC loadings > 0.90) (FB = faba bean; CP = chickpea; WL = whole lentil; SP = split pea; PB = pinto bean).

	Peak area (mean ± standard deviation)					
KI_{exp}	Control	FB	CP	WL	SP	PB
MXT-5-FID1						
505	3912 ± 225[e]	13965 ± 698[c,d]	12354 ± 1146[d]	19977 ± 620[a,b]	17241 ± 1755[b,c]	22886 ± 2551[a]
687	709 ± 54[e]	2169 ± 312[c,d]	1998 ± 166[d]	2700 ± 229[b,c]	3884 ± 598[a]	2819 ± 262[b]
704	822 ± 46[d]	1814 ± 158[c]	2206 ± 219[c]	2658 ± 261[b]	3828 ± 283[a]	1998 ± 174[c]
805	1282 ± 113[e]	5897 ± 742[d]	9567 ± 820[c]	10570 ± 1210[a,b]	11893 ± 1140[a]	8062 ± 591[c]
894	11 ± 24[d]	195 ± 16[c]	326 ± 24[b]	341 ± 30[a,b]	373 ± 26[a]	363 ± 21[a,b]
MXT-1701-FID2						
523	1762 ± 166[d]	10016 ± 449[b,c]	10006 ± 940[c]	17940 ± 2743[a]	13154 ± 1557[a,b]	18310 ± 2572[a]
677	38 ± 335[d]	254 ± 33[c]	337 ± 35[b,c]	348 ± 25[b]	608 ± 80[a]	291 ± 29[b,c]
689	0 ± 0[d]	398 ± 26[c]	354 ± 37[c]	666 ± 69[a]	664 ± 68[a]	474 ± 37[b]
800	772 ± 153[d]	3256 ± 337[c]	3634 ± 447[b,c]	4320 ± 482b	6562 ± 650[a]	3780 ± 339[b,c]
897	1045 ± 121[e]	5489 ± 950[d]	9305 ± 753[b,c]	10203 ± 1249[a,b]	11554 ± 1210[a]	7602 ± 616[c]

Different letters in the same rom indicate statistically significant differences ($p < 0.05$).
